# Supplementary material for: Geometric representations of brain networks can predict the surgery outcome in temporal lobe epilepsy
Source: NPJ Syst Biol Appl. 2025 Jul 16;11:79. doi: 10.1038/s41540-025-00562-6 (PMC12267629; doi:10.1038/s41540-025-00562-6)
Supplement: Supplementary file 1 — Supplementary Information [file 41540_2025_562_MOESM1_ESM.pdf]

Geometric representations of brain networks can predict the surgery outcome in temporal lobe epilepsy

Martin Guillemaud, Alice Longhena, Louis Cousyn, Valerio Frazzini, Bertrand Mathon, Vincent Navarro, Mario Chavez

Supplementary Material

1 Euclidean vs hyperbolic embedding

To evaluate the added value of using hyperbolic geometry over a standard Euclidean representation, we conducted a comparative analysis using the same embedding-based pipeline. For each patient, both the pre- and post-surgery brain networks were constructed and separately embedded into either Euclidean or hyperbolic space.

Euclidean coordinates were derived using Laplacian eigenmaps, a nonlinear dimensionality reduction technique currently employed in graphs embedding algorithms [1]. To evaluate differences between favorable and unfavorable outcome groups, we computed node-wise discriminative scores for both embedding types using a permutation-based approach. Specifically, for each node, we calculated the distance—according to the appropriate geometric metric—between its pre- and post-surgical coordinates in the embedding space. These node-wise scores were then statistically compared between the two outcome groups using a permutation-based t-test. Nodes exhibiting statistically significant differences ($p < 0.05$) are highlighted in red.

As illustrated in the supplementary Fig. 1, only a single node located in the hemisphere contralateral to the site of surgery exhibits a statistically significant difference in the Euclidean embedding space. In contrast, seven nodes demonstrate significant differences in the hyperbolic embedding space ($p_{value} < 0.05$). These findings further support our conclusion that hyperbolic embeddings provide greater sensitivity in detecting outcome-related connectivity changes in brain networks.

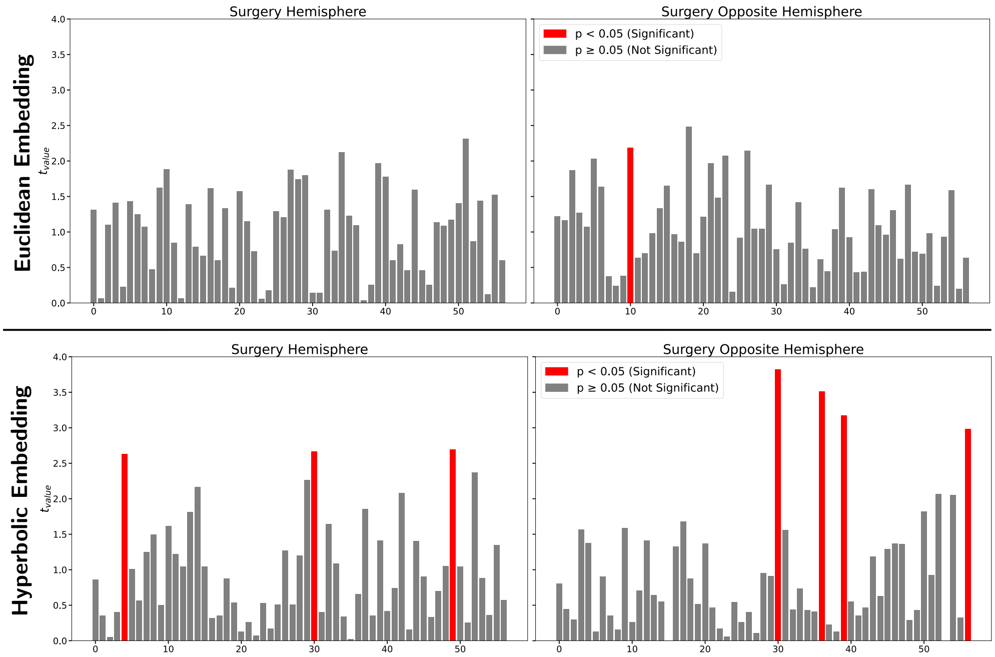

Supplementary Figure 1 Comparison between Euclidean and hyperbolic embeddings. Nodes with statistically significant differences between outcome groups (favorable vs. unfavorable) are highlighted in red ($p_{value} < 0.05$).

2 Average HypDisp values: comparison of groups' outcome

To further illustrate the differences in *HypDisp* profiles between patient groups, we computed and visualized group-level average disks for favorable and unfavorable outcomes, separately for left and right hemisphere surgeries. In these visualizations, each pixel within a disk represents the mean *HypDisp* value at that specific location, averaged across all patients in the corresponding group.

In addition to the average maps, we generated statistical comparison maps using a two-sample t-test. In these maps, non-grey regions indicate pixels with p_{values} below 0.05, thereby highlighting statistically significant differences between the groups. The first row of Supplementary Fig. 2 displays the results for patients who underwent left hemisphere surgery, where the group difference in average *HypDisp* values is visually prominent. The second row shows the results for right hemisphere surgeries; although the differences in mean values are less visually apparent, the t-test reveals localized regions of significance. This underscores the added value of the statistical comparison, which captures differences not only in central tendency but also in variability across patient groups.

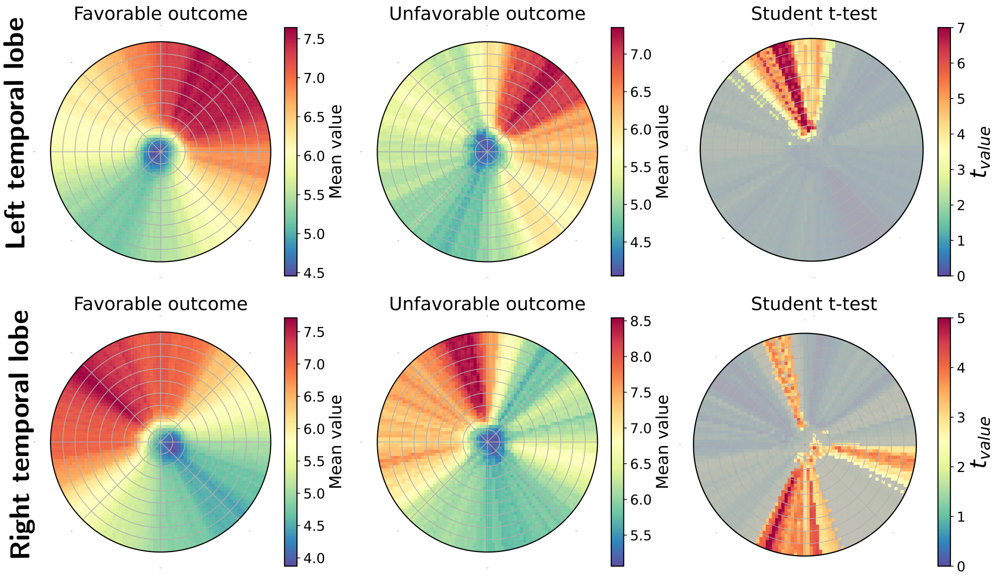

Supplementary Figure 2 Group-level *HypDisp* maps for patients with favorable (first column) and unfavorable (second column) outcomes, shown separately for left- (top row) and right-hemisphere (bottom row) surgeries. The third column displays pixel-wise two-sample t-tests between groups; non-grey regions denote statistically significant differences ($p < 0.05$).

References

[1] Von Luxburg, U. A tutorial on spectral clustering. *Statistics and computing* **17**, 395–416 (2007). URL https://doi.org/10.1007/s11222-007-9033-z.
